# Therapeutic Potential of *Lavandula stoechas* Aqueous Extract in Managing Diabetes and Its Complications: Insights From Biochemical and In Silico Studies

**DOI:** 10.1002/fsn3.70469

**Published:** 2025-06-20

**Authors:** Amal Elrherabi, Fahd A. Nasr, Ayoub Farihi, Mohamed Bouhrim, El Hassania Loukili, Mounia Driouech, Nour elhouda Daoudi, Mohammed Al‐zharani, Ashraf Ahmed Qurtam, Mohamed Bnouham

**Affiliations:** ^1^ Laboratory of Bioresources, Biotechnology, Ethnopharmacology, and Health, Faculty of Sciences Oujda (FSO) University Mohammed First (UMP) Oujda Morocco; ^2^ Biology Department, College of Science Imam Mohammad Ibn Saud Islamic University (IMSIU) Riyadh Saudi Arabia; ^3^ Laboratory of Pharmacology, Pharmacokinetics and Clinical Pharmacy, Faculty of Pharmacy University of Lille Lille France; ^4^ Laboratory of Biological Engineering, Team of Functional and Pathological Biology, Faculty of Sciences and Techniques Beni Mellal University Sultan Moulay Slimane Beni Mellal Morocco; ^5^ Euromed University of Fes Fès Morocco

**Keywords:** antihyperglycemic, diabetes, dyslipidemia, in silico analysis, *Lavandula stoechas*, phytochemicals

## Abstract

Diabetes mellitus is a chronic metabolic disorder characterized by persistent hyperglycemia, commonly associated with dyslipidemia, oxidative stress, and complications affecting multiple organs, including the kidneys and liver. These complications result from prolonged insulin deficiency or resistance, leading to impaired glucose and lipid metabolism, as well as increased oxidative damage. Effective diabetes management requires not only glycemic control but also addressing associated metabolic and oxidative imbalances. Natural therapies with multi‐target actions, such as the aqueous extract of *Lavandula stoechas* (AqLs), have gained attention for their potential to mitigate both hyperglycemia and related complications. This study investigated the therapeutic efficacy of AqLs in managing diabetes and its complications. The aerial parts of *L. stoechas* were collected, dried, and prepared using the decoction method. Alloxan‐induced diabetic rats received AqLs at doses of 150 mg/kg and 300 mg/kg for 4 weeks. Biochemical parameters, including blood glucose, lipid profiles, liver glycogen, and markers of kidney and liver function, were assessed. In silico ADME and toxicity assessments were performed to examine the pharmacokinetic behavior and safety of the extract's phytochemicals. AqLs significantly reduced blood glucose levels, improved lipid parameters, and prevented body weight loss in diabetic rats. It also enhanced liver glycogen levels and ameliorated markers of kidney and liver dysfunction. In silico analysis showed that compounds such as naringin and syringic acid demonstrated favorable pharmacokinetics and safety, supporting their potential as orally administered therapeutic agents. These findings suggest that *L. stoechas* aqueous extract exerts significant antihyperglycemic, lipid‐lowering, and organ‐protective effects, making it a promising natural therapeutic agent for diabetes management.

## Graphical abstract

The aqueous extract of *Lavandula stoechas* (AqLs) significantly reduced blood glucose and improved lipid profiles in alloxan‐induced diabetic rats. It also enhanced liver glycogen and protected against liver and kidney dysfunction. In silico analysis of key phytochemicals supported their pharmacokinetic potential and safety, highlighting *L. stoechas* as a promising multi‐target natural therapy for diabetes management.

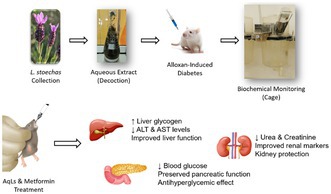

## 1 Introduction

Type 2 diabetes mellitus (T2DM) is a metabolic disorder characterized by disturbances in carbohydrate, lipid, and protein metabolism. It arises due to insufficient insulin production, reduced insulin sensitivity, or both. Among the three primary types of diabetes, T2DM is the most prevalent, accounting for over 90% of cases, making it significantly more common than type 1 diabetes mellitus (T1DM) or gestational diabetes. Considerable progress has been made in recent years toward understanding its pathogenesis and progression. T2DM primarily results from a progressive decline in pancreatic β‐cell function, typically accompanied by insulin resistance in key tissues such as skeletal muscle, liver, and adipose tissue. Before reaching the stage of full‐blown diabetes, individuals often experience prediabetes, characterized by elevated blood glucose levels that fall below the diabetic threshold, significantly increasing the risk of developing T2DM (Abdul‐Ghani et al. 2006; DeFronzo 2009). Prediabetes is defined by the presence of one or more of the following conditions: elevated fasting plasma glucose (impaired fasting glucose, IFG), reduced glucose clearance following carbohydrate intake (impaired glucose tolerance, IGT), or elevated levels of glycated hemoglobin (HbA1c). Individuals with IFG have fasting blood glucose levels above the normal range but below the diagnostic threshold for diabetes. In contrast, IGT is associated with insulin resistance in muscle tissue and a delayed (second‐phase) insulin secretion in response to food intake. Those with IFG primarily exhibit hepatic insulin resistance along with an inadequate initial (first‐phase) insulin secretion (Abdul‐Ghani et al. 2006). As the disease progresses, insulin secretion becomes insufficient to regulate glucose levels effectively, leading to persistent hyperglycemia. Individuals with T2DM are often obese or have an elevated body fat percentage, particularly concentrated in the abdominal area. In this condition, adipose tissue contributes to insulin resistance through multiple inflammatory mechanisms, including excessive release of free fatty acids (FFAs) and dysregulated adipokine production. The global rise in obesity, sedentary lifestyles, high‐calorie diets, and an aging population are the main drivers of the T2DM epidemic, resulting in a fourfold increase in its incidence and prevalence (Chatterjee et al. 2017; Zhou et al. 2016). Several organs play a crucial role in the development of T2DM, including the pancreas (β‐cells and α‐cells), liver, skeletal muscle, kidneys, brain, small intestine, and adipose tissue. Emerging research highlights the significance of adipokine imbalances, chronic inflammation, and disturbances in gut microbiota in the pathogenesis of the disease. Additionally, immune system dysregulation and inflammatory processes have been identified as major contributors to the progression of T2DM (DeFronzo 2009). Epidemiological studies reveal alarming trends, pointing to a substantial future burden of T2DM. According to the International Diabetes Federation (IDF), diabetes caused 4.2 million deaths in 2019, and affected 463 million adults aged 20–79. Projections estimate that this number could rise to 700 million by 2045, underscoring the urgent need for effective prevention and management strategies (Gæde et al. 2003).

Metformin is a primary oral antidiabetic drug frequently prescribed to manage type 2 diabetes. Both metformin and phenformin belong to the guanidine derivative class. Phenformin was isolated in the 1920s from extracts of *Galega officinalis*, commonly known as French lilac. Historically, isoamylene guanidine, also referred to as galegine, had been used for centuries in the treatment of diabetes (Bailey and Day 1989; Liu et al. 2015). Metformin lowers blood glucose by inhibiting hepatic gluconeogenesis and enhancing insulin sensitivity. Additionally, it promotes weight loss, improves lipid and liver function profiles, modulates inflammatory responses, and may reduce cancer risk (Hossain and Pervin 2018). Metformin is generally well tolerated but can cause gastrointestinal side effects, such as nausea and diarrhea, which are usually temporary. In rare cases, it may lead to lactic acidosis, particularly in patients with impaired kidney or liver function. Approximately 4% of patients discontinue treatment due to adverse effects (Hossain and Pervin 2018).

Despite the side effects associated with conventional antidiabetic drugs, traditional medicinal plants, rich in bioactive compounds, offer a natural alternative with notable antidiabetic properties and are generally considered safe, including flavonoids, alkaloids, phenolics, and tannins, which enhance pancreatic function by stimulating insulin secretion or reducing glucose absorption in the intestines (Kooti et al. 2016). Research indicates that around 410 medicinal plants have been experimentally validated for their antidiabetic properties, although the full mechanisms of action have been clearly identified for only 109 of them. Numerous plant extracts have demonstrated the ability to influence key metabolic pathways, including glycolysis, gluconeogenesis, the Krebs cycle, glycogen metabolism, insulin synthesis and release, cholesterol biosynthesis, as well as carbohydrate digestion and absorption (Prabhakar and Doble 2008).

The plant under study, *Lavandula stoechas* , a member of the Lamiaceae family, has demonstrated notable antidiabetic properties through multiple mechanisms. Our previous research confirmed its ability to reduce postprandial hyperglycemia by inhibiting glucose absorption and key digestive enzymes, as well as enhancing insulin function and glucose uptake. Additionally, *L. stoechas* exhibited strong antioxidant, antiglycation, and anti‐inflammatory activities, along with pancreatic lipase inhibition, suggesting its potential in managing diabetic complications. HPLC analysis identified phenolic compounds contributing to its bioactivity, while in silico ADME‐Tox predictions supported a favorable pharmacokinetic profile. Building on these findings (Elrherabi et al. 2023a, 2023b, 2024).

This study aims to evaluate the antidiabetic potential of the aqueous extract of *L. stoechas* in an alloxan‐induced diabetic rat model. Specifically, it examines the extract's effects on key metabolic and physiological markers of diabetes, including glycemic control, lipid profile, renal and hepatic function, and body weight regulation. The study also compares the extract's efficacy with that of standard antidiabetic treatments, providing insights into its potential therapeutic application in diabetes management. This research is novel in exploring the use of a natural aqueous extract of the relatively understudied *L. stoechas* as a complementary or alternative treatment for diabetes. By examining its antioxidant, anti‐inflammatory, and glucose‐regulating properties, this study offers new perspectives on the plant's role in managing diabetes and its potential as an adjunct to current treatments.

## 2 Materials and Methods

### 2.1 Plant Extraction

In November 2023, *L. stoechas* samples were collected from Tafoughalt in Oriental Morocco (34.805497, −2.406913). A botanist authenticated the plant specimen, which was archived in the herbarium of the Faculty of Sciences at Mohamed First University, Oujda, under reference number HUMPOM77. To prepare the plant extract, the aerial parts of *L. stoechas* were carefully washed with distilled water to eliminate impurities and then dried at 40°C in an oven. The dried plant material was finely ground into a powder. For extraction, 80 g of the powder were boiled in 800 mL of distilled water for 20 min using the decoction method. The mixture was filtered, and the resulting aqueous extract was concentrated by drying at 40°C to produce a powdered form. The extraction yield was approximately 100 mg per gram of dried plant material. This meticulous preparation process ensured the purity and quality of the *L. stoechas* extract, making it suitable for further experimental use.

### 2.2 Induction of Diabetes

Diabetes was induced in fasted rats by a single intraperitoneal (i.p.) injection of freshly prepared alloxan monohydrate at a dose of 120 mg/kg, dissolved in phosphate–citrate buffer (pH 4.5). After the injection, rats were allowed to feed *ad libitum* to promote alloxan uptake by pancreatic β‐cells, resulting in their selective destruction.

### 2.3 Animal Grouping

The animals were randomly divided into five groups, each containing six rats: the Control Group, consisting of healthy rats gavaged with distilled water (10 mL/kg); the Alloxan Group, comprising diabetic rats gavaged with distilled water (10 mL/kg); the Alloxan + AqLs (150 mg/kg) Group, which included diabetic rats gavaged with an aqueous extract of *L. stoechas* (150 mg/kg) dissolved in 10 mL of distilled water; the Alloxan + AqLs (300 mg/kg) Group, involving diabetic rats gavaged with an aqueous extract of *L. stoechas* (300 mg/kg) dissolved in 10 mL of distilled water; and the Metformin Group, consisting of diabetic rats gavaged with metformin (2 mg/kg) dissolved in 10 mL of distilled water.

### 2.4 Treatment Procedure

The treatment was administered orally once daily for a period of 30 consecutive days. Throughout the study, blood glucose levels were measured weekly. Body weight, daily food intake, water consumption, and urine output were recorded for each animal both before and after the treatment period. At the end of the experiment, the rats were fasted for 14 h, anesthetized using isoflurane, and subsequently sacrificed. Blood samples were then collected for biochemical analysis.

### 2.5 Hepatic Glycogen Assay

Hepatic glycogen extraction and quantification were performed according to the method described by Ong and Khoo with some modifications (Ong and Khoo 2000). Liver samples weighing between 0.3 and 0.5 g were first minced and homogenized in 2 mL of 30% potassium hydroxide, then boiled at 100°C for 30 min. To precipitate glycogen, the mixture was treated twice with 4 mL of 95% ethanol. After each treatment, the mixture was stored at 4°C for 30 min. The mixture was then centrifuged at 3000 rpm for 15 min. The precipitate was washed with 8 mL of 95% ethanol and centrifuged again at 3000 rpm for 15 min. Finally, the glycogen was dissolved in 1 mL of distilled water. The glycogen concentration was determined using the anthrone reagent, and absorbance was measured at a wavelength of 625 nm.

#### 2.5.1 Preparation of Anthrone Reagent

The anthrone reagent was prepared using a combination of anthrone, thiourea, and 72% sulfuric acid. Specifically, 500 mg of anthrone (molecular weight: 194.23 g/mol) and 10 g of thiourea (molecular weight: 76.12 g/mol) were used per liter of the reagent. The sulfuric acid, with a molecular weight of 98.08 g/mol, was added at a concentration of 72% to complete the preparation.

#### 2.5.2 Glycogen Standard Curve

The glycogen standard curve was established using glycogen extracted and purified from rabbit liver (Sigma Aldrich). A stock solution of glycogen at a concentration of 1 mg/mL was prepared. From this stock solution, different standard solutions were prepared at concentrations of 500, 250, 125, 63, 32, 16, 8, and 4 μg/mL. These standard solutions were used as references to quantify the glycogen concentration in the test samples.

### 2.6 Biochemical Assays

Clinical diagnostic kits were utilized in conjunction with the Architect Abbott ci 8200 analyzers to evaluate separated plasma samples for blood glucose levels, total cholesterol (TC), triglycerides (TG), urea, uric acid, creatinine, aspartate aminotransferase (AST), alanine aminotransferase (ALT), and urinary glucose levels (Ercin et al. 2016).

### 2.7 In Silico Prediction of Toxicity

Early evaluation of the toxicological properties of chemical structures is essential in the drug discovery process (Ahmed and Alkali 2019). In silico toxicity models aim to predict the toxic effects of chemical compounds, thereby minimizing time, cost, and the use of animal testing (Banerjee and Ulker 2022). The toxicity of the compounds previously identified in the aqueous extract of *L. stoechas* (Elrherabi et al. 2024) was evaluated using the online platform ProTox‐II (Palanisamy et al. 2021). This tool employs a statistical algorithm to analyze the chemical structure of a substance by comparing it to a database of known toxic substances. It also provides information on LD_50_ values, toxicity class, and various toxicological parameters such as hepatotoxicity, nephrotoxicity, cytotoxicity, and immunotoxicity. The predicted assessment criteria results are expressed categorically (active or inactive) (Yeni and Rachmania 2023).

### 2.8 ADME Studies

With advancements in computational tools, predicting ADME properties has become increasingly important (Hbika et al. 2025). These tools assess a molecule's ability to cross cell membranes, its interactions with transporters and enzymes involved in absorption and excretion, and its metabolic stability. Evaluating the pharmacokinetic properties of a compound, including absorption, distribution, metabolism, and excretion (ADME), is essential for understanding its behavior in the body (Laaraj, Tikent, et al. 2024), as these processes dictate its journey from administration to elimination. In this study, we employed the SwissADME platform (available at www.swissadme.ch, accessed on February 14, 2025) (Farihi et al. 2023). To perform a comprehensive analysis of the phytochemicals we have already identified in the aqueous extract of *L. stoechas* (Elrherabi et al. 2024). This approach allowed us to assess their pharmacokinetic properties in detail and evaluate their potential therapeutic applications.

### 2.9 Statistical Analyses

All experimental data were expressed as mean ± standard deviation (SD). Statistical analysis was carried out using GraphPad Prism version 5.0. To determine the significance of differences between experimental groups, one‐way analysis of variance (ANOVA) was performed, followed by Tukey's multiple comparisons post hoc test to identify specific intergroup differences. A *p*‐value < 0.05 was considered statistically significant.

## 3 Results

### 3.1 Effect on Food Intake, Water Intake, and Urinary Volume

**FIGURE 1:**
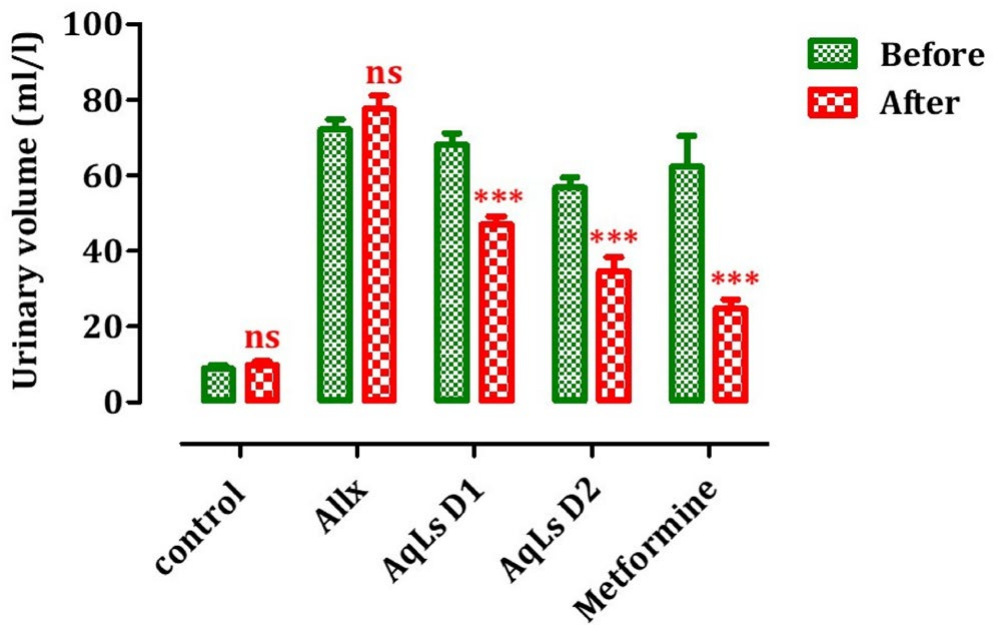
Effect of oral administration of the aqueous extract of *L. stoechas* at D1 = 150 and D2 = 300 mg/kg on urinary volume in alloxan‐induced diabetic rats (Allx). ***p < 0.001, significantly different from pre‐treatment. Allx, Diabetic control group.

**FIGURE 2:**
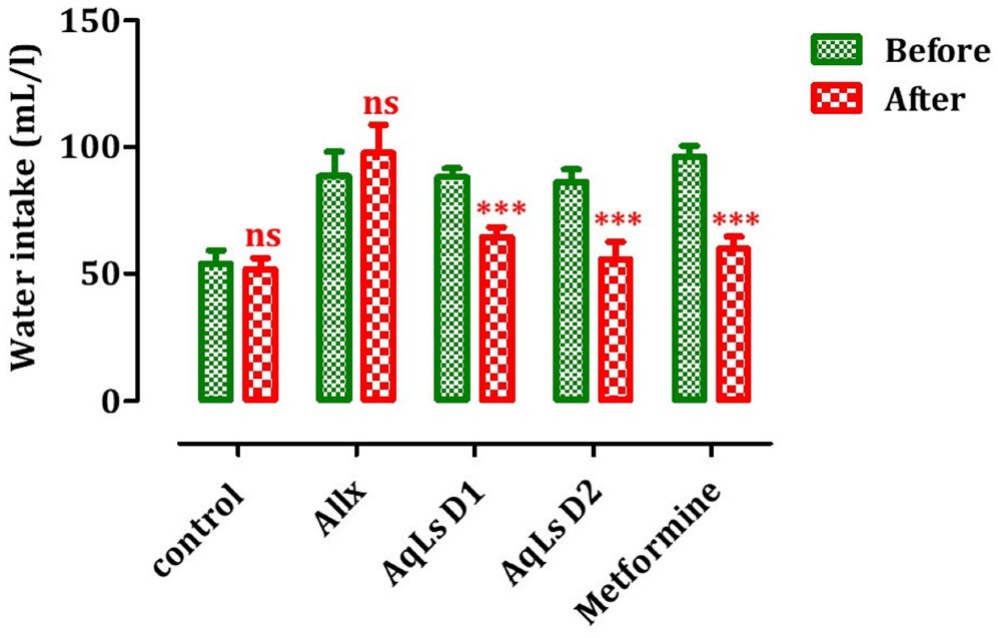
Effect of oral administration of the aqueous extract of *L. stoechas* at D1 = 150 and D2 = 300 mg/kg on water intake in alloxan‐induced diabetic rats (Allx). ***p < 0.001, significantly different from pre‐treatment. Allx, Diabetic control group.

**FIGURE 3:**
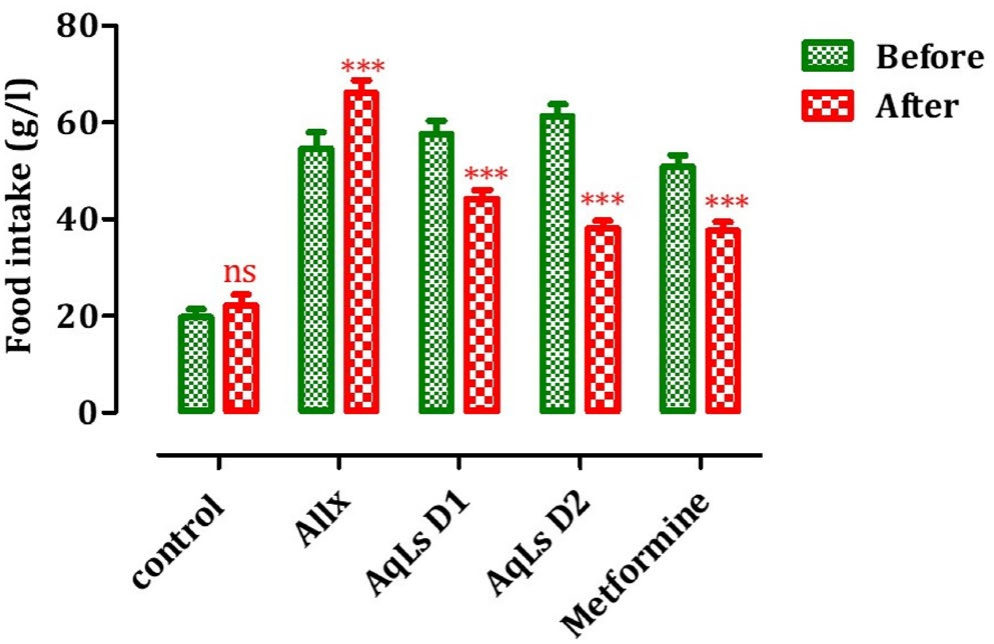
Effect of oral administration of the aqueous extract of *L. stoechas* at D1 = 150 and D2 = 300 mg/kg on food intake in alloxan‐induced diabetic rats (Allx). ***p < 0.001, significantly different from pre‐treatment. Allx, Diabetic control group.

The results highlighting the effect of AqLs on diabetes symptoms are presented in Figures 1, 2, 3. Indeed, the administration of both doses of AqLs, or 150 mg/kg of metformin, significantly reduced (*p* < 0.001) urinary volume in diabetic rats compared to the pre‐treatment period. Intraperitoneal injection of alloxan led to an increase in water consumption in the rats. Treatment of these rats with 150 or 300 mg/kg of AqLs, or 150 mg/kg of metformin for 4 weeks, significantly reduced (*p* < 0.001) water consumption in the diabetic rats compared to the pre‐treatment period (Figure 2). Additionally, alloxan caused a significant variation (*p* < 0.001) in food intake in the treated diabetic rats compared to the control group (Figure 3).

### 3.2 Effect on Body Weight

**FIGURE 4:**
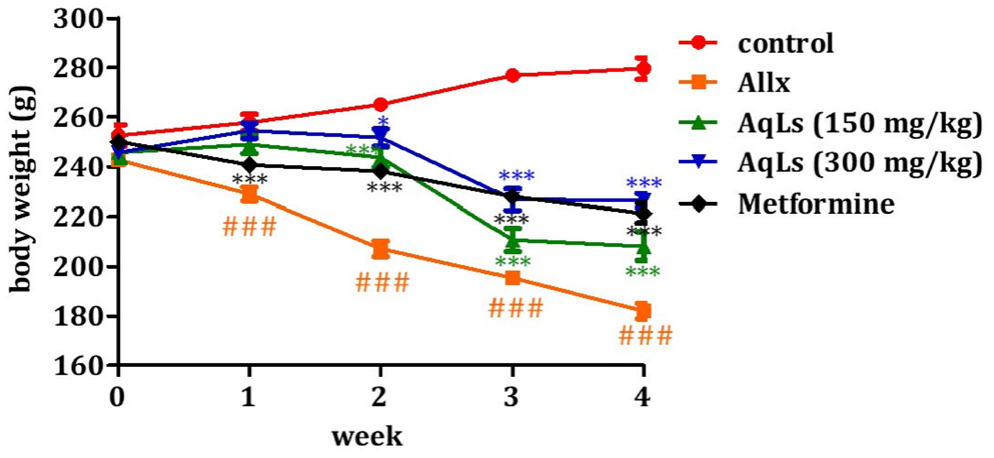
Effect of oral administration of the aqueous extract of *L. stoechas* at D1 = 150 mg/kg and D2 = 300 mg/kg on body weight gain in diabetic rats is presented. ^###^ *p* < 0.001, significantly different from the normal group. ***p < 0.001, *p < 0.05, significantly different from pre‐treatment. Allx, Diabetic control group.

Figure 4 illustrates the changes in body weight among normal and diabetic rats over the four‐week experimental period. Untreated diabetic rats (Allx) exhibited a significant loss in body weight (*p* < 0.001), declining from 242.6 ± 1.31 g to 182 ± 2.78 g, in contrast to the stable weight of the control group. In contrast, oral administration of the aqueous extract of *L. stoechas* (AqLs) at both tested doses significantly mitigated weight loss in diabetic rats (*p* < 0.001). For the 150 mg/kg dose, body weight changed from 245.56 ± 3.76 g to 208.1 ± 5.16 g, while the 300 mg/kg dose resulted in an increase from 245.7 ± 1.94 g to 226.32 ± 2.58 g. Similarly, treatment with metformin (150 mg/kg) significantly counteracted weight loss, with weight increasing from 250.16 ± 2.36 g to 221.34 ± 3.48 g (*p* < 0.001), compared to untreated diabetic rats.

### 3.3 Effect on Blood Glucose Levels

**FIGURE 5:**
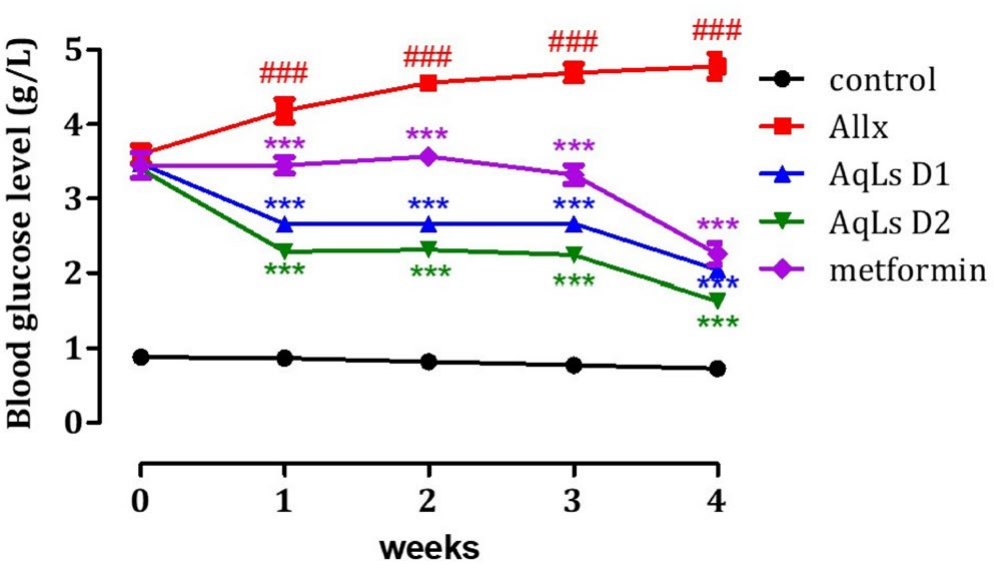
Variation in blood glucose levels before and after 4 weeks of diabetes induction by alloxan. Values are presented as mean ± SEM (*n* = 6). ***p < 0.001: Groups compared to the alloxan group (Allx). ^###^ *p* < 0.001: Groups compared to the normal group.

Intraperitoneal injection of alloxan at a single concentration (120 mg/kg) resulted in a significant increase (*p* < 0.001) in fasting blood glucose levels in rats (Allx group) over 30 days compared to normal rats (control group) (Figure 5). Additionally, daily administration of 150 mg/kg of AqLs to diabetic rats induced a decrease in their fasting blood glucose levels after 4 weeks, with a significant effect (*p* < 0.001) throughout this period. In comparison, diabetic rats receiving 300 mg/kg of AqLs also showed a significant decrease (*p* < 0.001) in fasting blood glucose levels during the treatment period compared to diabetic control rats (Allx group). Diabetic rats treated with glibenclamide (2 mg/kg) also exhibited a substantial decrease in fasting blood glucose levels (*p* < 0.001) (Table 1).

**TABLE 1:** Effect of the aqueous extract of *L. stoechas* on blood glucose levels in Allx‐diabetic rats.

Blood glucose level (g/L)					
Groups	Before treatment	First week	Second week	Third week	Fourth week
Control	0.88 ± 0.02	0.87 ± 0.01	0.82 ± 0.01	0.77 ± 0.00	0.73 ± 0.00
Allx	3.59 ± 0.10	4.18 ± 0.14	4.55 ± 0.07	4.69 ± 0.10	4.78 ± 0.15
Allx + AqLs (150 mg/kg)	3.47 ± 0.06	2.65 ± 0.01	2.66 ± 0.01	2.66 ± 0.01	2.04 ± 0.01
Allx + AqLs (300 mg/kg)	3.39 ± 0.08	2.29 ± 0.02	2.31 ± 0.06	2.24 ± 0.02	1.63 ± 0.02
Allx + metformin (150 mg/kg)	3.44 ± 0.15	3.44 ± 0.09	3.56 ± 0.06	3.32 ± 0.11	2.25 ± 0.13

### 3.4 Effect on Liver Glycogen

**FIGURE 6:**
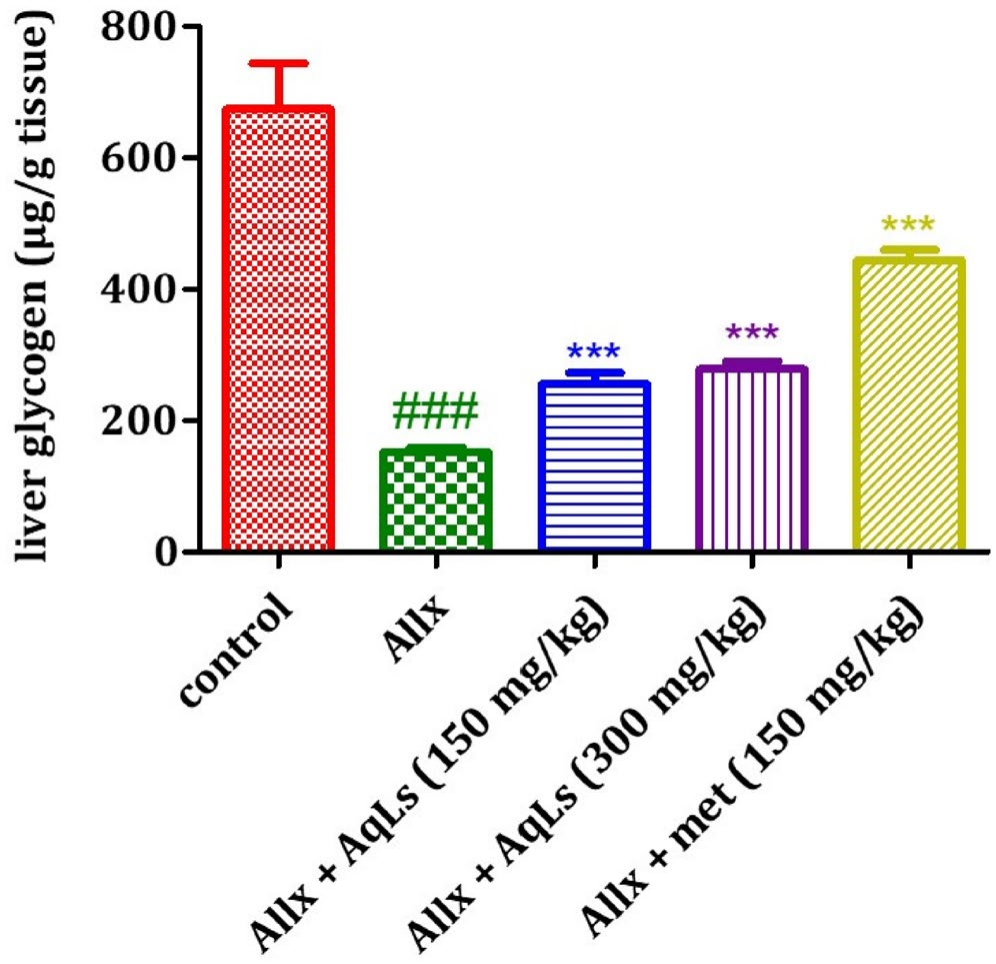
Effect of oral administration of the aqueous extract of *L. stoechas* at 150 and 300 mg/kg on liver glycogen levels in diabetic rats. The bar graph represents the mean ± SEM. ^###^Significantly different from the control group; ***p < 0.001 significantly.

The effect of AqLs on liver glycogen levels in diabetic rats is shown in Figure 6. The results indicate that the glycogen levels in diabetic rats (Allx group) are significantly lower (*p* < 0.001) compared to normal rats (control group). Furthermore, the administration of AqLs (at doses of 150 and 300 mg/kg) as well as metformin (at a dose of 150 mg/kg) significantly increased liver glycogen levels (*p* < 0.001) compared to diabetic rats in the Allx group.

### 3.5 Effect on Triglyceride and Total Cholesterol Levels

**FIGURE 7:**
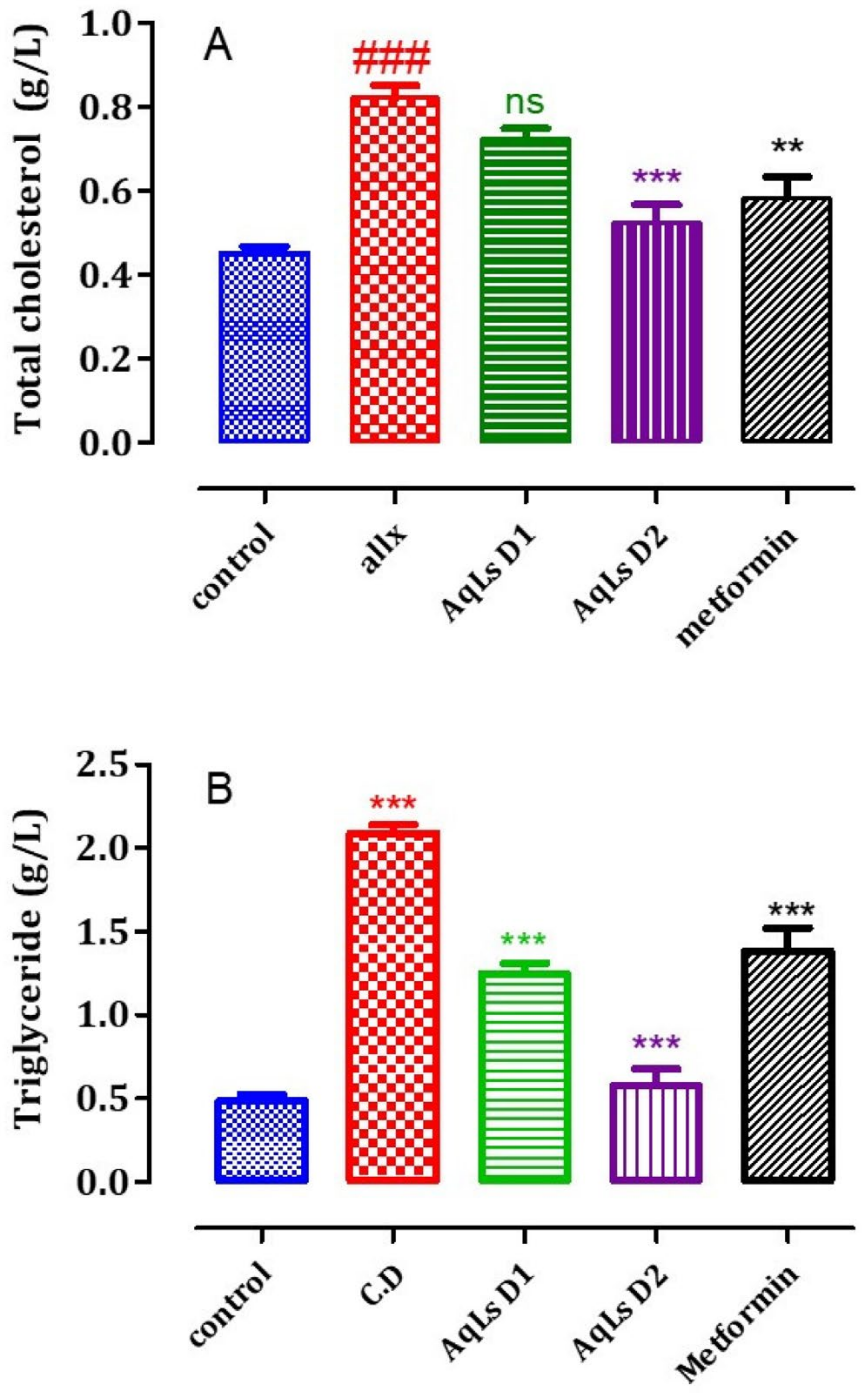
Effect of oral administration of *L. stoechas* aqueous extract at 150 and 300 mg/kg on total cholesterol (A) and triglyceride (B) levels in diabetic rats. The bar graph represents the mean ± SEM. ^###^ *p* < 0.001 significantly different from the control group. ***p < 0.001, *p < 0.05, significantly different from pre‐treatment.

The impact of AqLs on plasma levels of total cholesterol (TC) and triglycerides (TGs) in diabetic rats is shown in Figure 7. The data reveal that plasma TC and TG levels in diabetic rats (Allx group) are significantly higher (*p* < 0.001) than those in normal rats (control group). Administration of AqLs (at a dose of 300 mg/kg) significantly reduced plasma TC levels (*p* < 0.001) and TG levels (*p* < 0.001) at both doses of 150 mg/kg and 300 mg/kg. Additionally, metformin also significantly reduced plasma TC and TG levels (*p* < 0.001) compared to diabetic control rats in the Allx group.

### 3.6 Effect on Plasma Urea, Creatinine, and Uric Acid Levels

**FIGURE 8:**
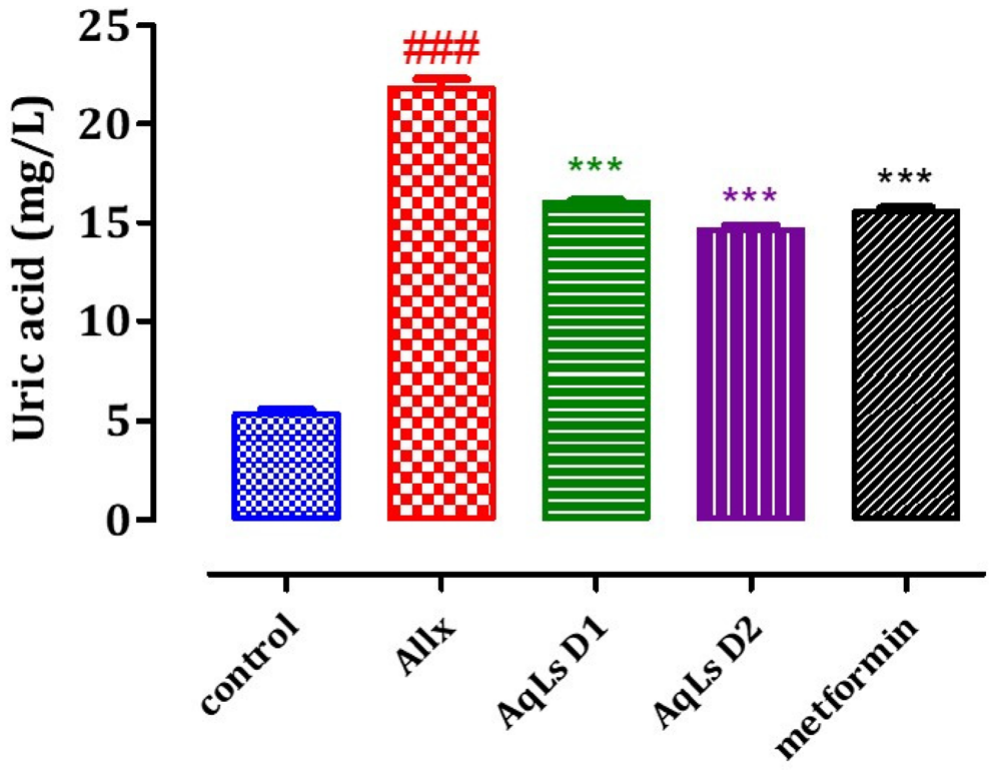
Effect of oral administration of *L. stoechas* aqueous extract at 150 and 300 mg/kg on serum uric acid levels in Allx‐diabetic rats. The bar graph represents the mean ± SEM. ^###^ *p* < 0.001 significantly different from the control group. ***p < 0.001 significantly different from the Allx group. Allx, Alloxan.

**FIGURE 9:**
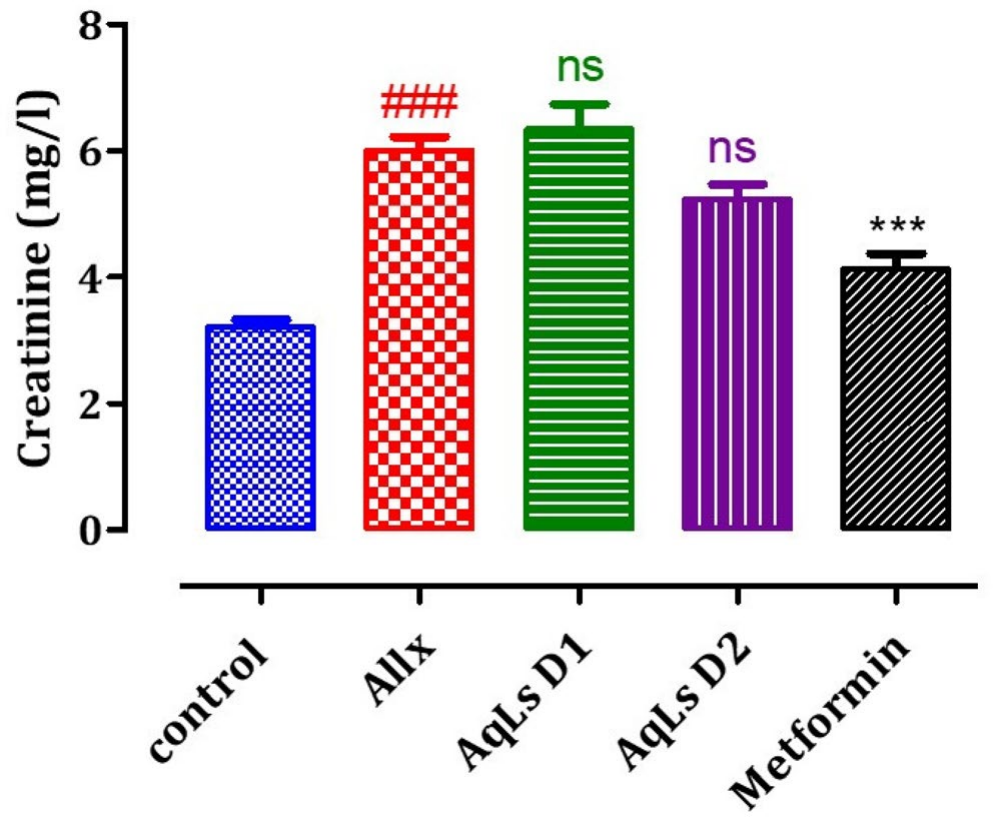
Effect of oral administration of *L. stoechas* aqueous extract at 150 and 300 mg/kg on creatinine levels in Allx‐diabetic rats. The bar graph represents the mean ± SEM. ^###^ *p* < 0.001 significantly different from the control group. ***p < 0.001 significantly different from the Allx group. Allx, Alloxan.

**FIGURE 10:**
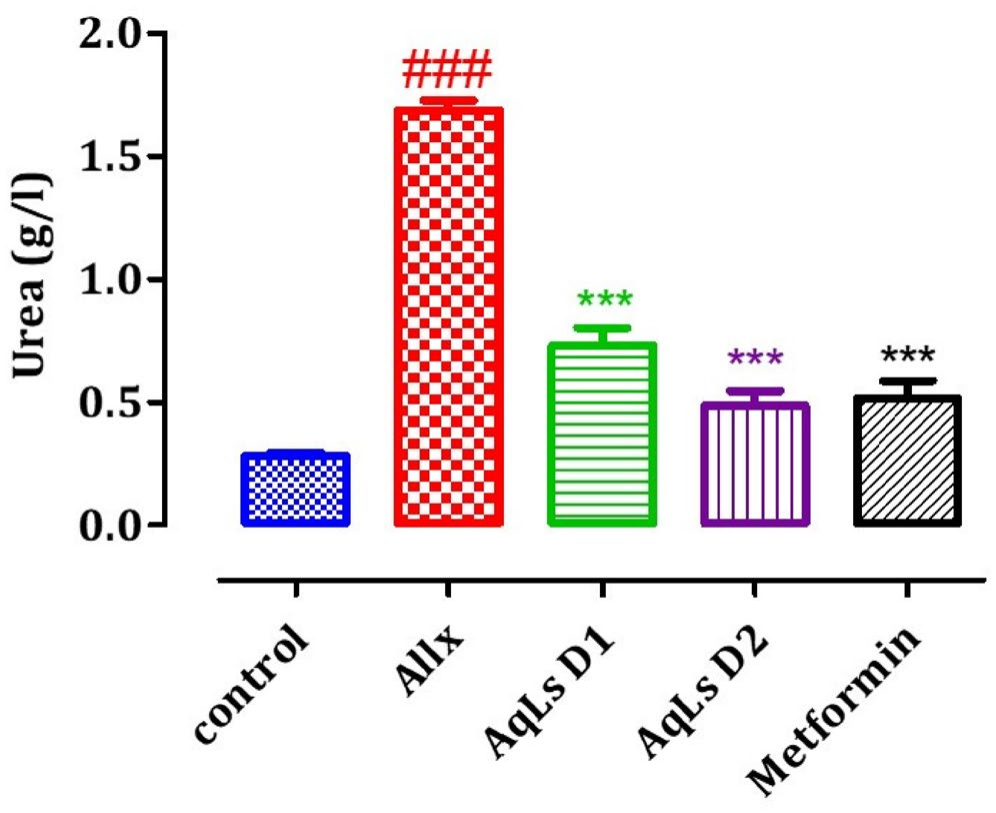
Effect of oral administration of *L. stoechas* aqueous extract at 150 and 300 mg/kg on urea levels in Allx‐diabetic rats. The bar graph represents the mean ± SEM. ^###^ *p* < 0.001 significantly different from the control group. ***p < 0.001 significantly different from the Allx group. Allx, Alloxan.

The effect of AqLs administration on plasma uric acid, creatinine, and urea levels in diabetic rats is shown in Figures 8, 9, 10. The results indicate that plasma levels of uric acid, creatinine, and urea are significantly higher in diabetic rats from the Allx control group compared to normal rats from the control group (*p* < 0.001). Administration of AqLs at doses of 150 and 300 mg/kg resulted in a significant decrease in uric acid (*p* < 0.001) and urea (*p* < 0.001) compared to diabetic rats from the Allx control group. Additionally, administration of metformin at a dose of 150 mg/kg also resulted in a significant reduction in uric acid, creatinine, and urea compared to diabetic rats from the Allx control group (*p* < 0.001).

### 3.7 Effect on Plasma Levels of Alanine Aminotransferase and Aspartate Aminotransferase

**FIGURE 11:**
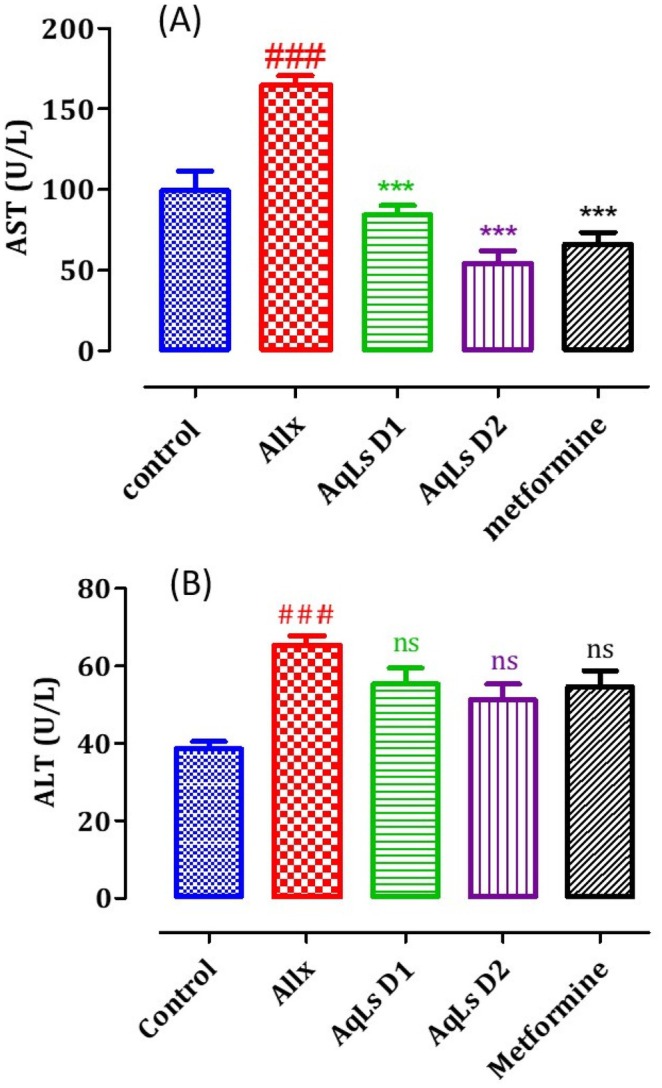
Effect of oral administration of *L. stoechas* aqueous extract at 150 and 300 mg/kg on plasma levels of AST (A) and ALT (B) in Allx‐diabetic rats. The bar graph represents the mean ± SEM. ^###^ *p* < 0.001 compared to the control group. ***p < 0.001 compared to the Allx group. Allx, Alloxan.

The impact of AqLs administration on plasma levels of aspartate aminotransferase (AST) and alanine aminotransferase (ALT) in diabetic rats is illustrated in Figure 11. The results reveal a significant increase (*p* < 0.001) in plasma ALT and AST levels in the diabetic rats of the Allx control group compared to normal rats. Administration of AqLs at doses of 150 and 300 mg/kg resulted in a significant reduction in AST levels compared to the diabetic rats in the Allx control group (*p* < 0.001). Similarly, metformin administration at a dose of 150 mg/kg also induced a significant decrease in AST levels compared to the diabetic rats in the Allx control group (*p* < 0.001).

### 3.8 Prediction of Compounds Toxicity

The in silico toxicity assessment of the main phytocompounds identified in the aqueous extract of *L. stoechas* highlights significant variations in terms of organ and systemic toxicity. The analysis reveals that all compounds, except cinnamic acid, are considered non‐hepatotoxic. However, nephrotoxicity is predicted for all compounds, with probabilities ranging from 0.55 to 0.77. Regarding cytotoxicity, the majority of compounds are classified as non‐cytotoxic, indicating a generally low risk of cellular toxicity; however, naringin (*p* = 0.99) is an exception, indicating a potential risk of cytotoxicity for this molecule. Immunotoxicity analysis indicated that only ferulic acid was predicted to be immunotoxic (*p* = 0.91), whereas other compounds were deemed safe for the immune system. Regarding acute toxicity, catechin exhibited the lowest toxicity (toxicity class 6, LD_50_ = 10,000 mg/kg), while the remaining compounds fell into toxicity classes 4 and 5, with LD_50_ values ranging from 1700 to 2850 mg/kg (Table 2).

**TABLE 2:** In silico prediction of organ toxicity and toxicological endpoints of phytochemicals from the aqueous extract of *Lavandula stoechas* .

Compounds	Organ toxicity		Toxicity end points		Class	LD_50_ mg/kg
	Hepatotoxicity	Nephrotoxicity	Cytotoxicity	Immunotoxicity		
Ferulic acid	Inactive (*p* = 0.51)	Active (*p* = 0.62)	Inactive (*p* = 0.88)	Active (*p* = 0.91)	4	1772
Catechin	Inactive (*p* = 0.72)	Active (*p* = 0.62)	Inactive (*p* = 0.96)	Inactive (*p* = 0.84)	6	10,000
Syringic acid	Inactive (*p* = 0.58)	Active (*p* = 0.66)	Inactive (*p* = 0.97)	Inactive (*p* = 0.97)	4	1700
4‐hydroxybenzoic acid	Inactive (*p* = 0.52)	Active (*p* = 0.68)	Inactive (*p* = 0.86)	Inactive (*p* = 0.99)	5	2200
Naringin	Inactive (*p* = 0.81)	Active (*p* = 0.77)	Active (*p* = 0.99)	Inactive (*p* = 0.66)	5	2300
Cinnamic acid	Active (*p* = 0.54)	Active (*p* = 0.55)	Inactive (*p* = 0.95)	Inactive (*p* = 0.83)	5	2500
p‐coumaric acid	Inactive (*p* = 0.51)	Active (*p* = 0.66)	Inactive (*p* = 0.81)	Inactive (*p* = 0.91)	5	2850

Abbreviations: LD_50_, Lethal Dose 50; P, Probability.

### 3.9 ADME Analysis

**FIGURE 12:**
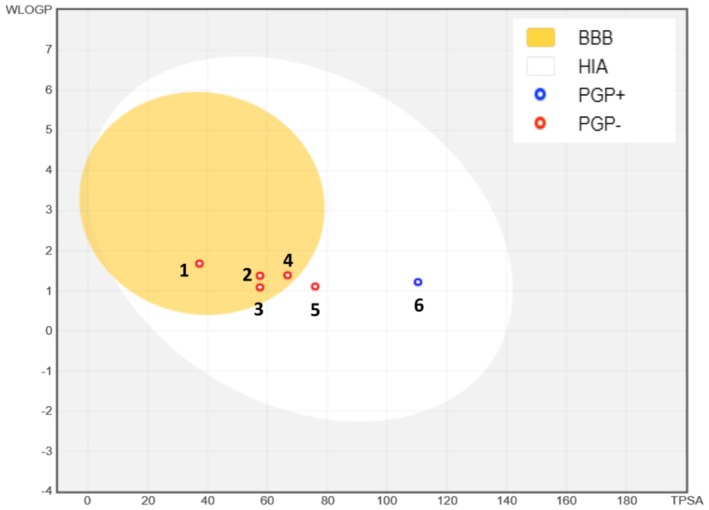
BOILED‐Egg Model of the GI absorption and BBB permeability of phytochemicals identified in the aqueous extract of Lavandula stoechas. (1) Cinnamic acid, (2) p‐coumaric acid, (3) 4‐Hydroxybenzoic acid, (4) Ferulic acid, (5) Syringic acid, and (6) Catechin. PGP‐: non‐substrate of P‐glycoprotein, PGP+: P‐glycoprotein substrate.

Computational models are instrumental in early pharmacokinetic screening, facilitating the rapid identification of promising drug candidates and accelerating the drug development process. In this study, all phytochemicals identified in the aqueous extract of *L. stoechas* L. satisfied Lipinski's Rule of Five, indicating good oral bioavailability potential—except for naringin, which violated three of the criteria. Notably, compounds such as ferulic acid (TPSA = 66.7 Å^2^, WLogP = 1.3), 4‐hydroxybenzoic acid (TPSA = 138.1 Å^2^, WLogP = 1.0), cinnamic acid (TPSA = 37.3 Å^2^, WLogP = 1.6), and p‐coumaric acid (TPSA = 57.5 Å^2^, WLogP = 1.3) demonstrated the ability to cross the blood–brain barrier (BBB) (Figure 12). All phytochemicals showed high predicted intestinal absorption, except for naringin (Table 3). Furthermore, all compounds—except catechin—were predicted as non‐substrates of P‐glycoprotein (PGP) (Figure 12). Importantly, none of the tested compounds were predicted to be inhibitors or substrates of cytochrome P450 enzymes, particularly CYP1A2 (Table 3).

**TABLE 3:** Evaluation of the pharmacokinetic properties (ADME) of phytochemicals identified in the aqueous extract of *Lavandula stoechas* (in silico).

Compounds	Physicochemical properties					Lipophilicity		Pharmacokinetics			Druglikeness	
	MW g/mol	HBA	HBD	TPSA Å^2^	ROTB	Mlog P	Wlog P	BBB permeation	GI absorption	CYP1A2 inhibitor	Lipinski violation	Verber violation
Catechin	290.2	6	5	110.3	1	0.24	1.2	No	High	No	0	0
Ferulic acid	194.1	4	2	66.7	3	1.0	1.3	Yes	High	No	0	0
4‐hydroxybenzoic acid	138.1	3	2	57.5	1	0.9	1.0	Yes	High	No	0	0
Syringic acid	198.1	5	2	75.9	3	0.4	1.1	No	High	No	0	0
Naringin	580.5	14	8	225.0	6	−2.7	−1.4	No	Low	No	3	1
Cinnamic acid	148.1	2	1	37.3	2	1.9	1.6	Yes	High	No	0	0
p‐coumaric acid	164.1	3	2	57.5	2	1.2	1.3	Yes	High	No	0	0

Abbreviations: BBB, blood–brain barrier; GI, gastrointestinal; HBA, hydrogen‐bond acceptors; HBD, hydrogen‐bond donors; MLogP, octanol/water partition coefficient; MW, molecular weight; ROTB, rotatable bonds; TPSA, topological polar surface area; WLogP, lipophilicity.

## 4 Discussion

The present study demonstrated that the aqueous extract of *L. stoechas* (AqLs) significantly improved diabetes‐related symptoms in alloxan‐induced diabetic rats. AqLs administration effectively reduced polyuria, polydipsia, and polyphagia, prevented body weight loss, and lowered fasting blood glucose levels. Furthermore, the extract improved hepatic glycogen storage, reduced plasma triglyceride and cholesterol levels, and ameliorated biochemical markers of renal and hepatic dysfunction. In our previous investigations, we elucidated some of the underlying antihyperglycemic mechanisms of AqLs, including its inhibitory activity against α‐amylase and α‐glucosidase, which slows carbohydrate digestion and reduces postprandial glucose spikes. Additionally, AqLs was shown to impair intestinal glucose absorption by inhibiting the sodium‐glucose cotransporter 2 (SGLT2), in a manner comparable to phlorizin (Elrherabi et al. 2023a). Building on our earlier findings, we demonstrated that the aqueous extract of *L. stoechas* (AqLs) exhibits significant antioxidant activity, effectively scavenging free radicals and inhibiting lipid peroxidation. Additionally, AqLs enhances glucose uptake in peripheral tissues, both independently and in synergy with insulin, indicating its ability to improve glucose utilization. The extract also showed notable antiglycation properties, reducing hemoglobin glycation in vitro, which highlights its potential to mitigate diabetes‐related complications (Elrherabi et al. 2023b). In our continued exploration of the aqueous extract of *L. stoechas* (AqLs), we identified key phenolic compounds such as naringin, syringic acid, and cinnamic acid through HPLC analysis, all known for their antioxidant and antidiabetic properties. The extract demonstrated significant antioxidant activity, effectively reducing iron and inhibiting lipid peroxidation, though slightly less potent than ascorbic acid. AqLs also exhibited pancreatic lipase inhibition, suggesting potential applications in managing obesity and dyslipidemia. Additionally, it showed antiglycation effects, preventing albumin glycation and the formation of advanced glycation end‐products (AGEs), as well as inhibiting β‐aggregation linked to protein misfolding. Furthermore, AqLs displayed anti‐inflammatory activity by inhibiting protein denaturation, akin to NSAIDs (Elrherabi et al. 2024). The significant reduction in fasting blood glucose levels observed in alloxan‐induced diabetic rats treated with the aqueous extract of *L. stoechas* (AqLs) can be attributed to its multifaceted mechanisms of action. These include the inhibition of carbohydrate‐digesting enzymes (α‐amylase and α‐glucosidase), reduced intestinal glucose absorption, enhanced peripheral glucose uptake, and improved insulin sensitivity. Additionally, the antioxidant, antiglycation, and anti‐inflammatory properties of AqLs, driven by its rich phenolic and flavonoid content, further contribute to its glucose‐lowering effects by protecting pancreatic β‐cells and mitigating diabetes‐related complications. Together, these mechanisms underscore the potential of AqLs as a natural therapeutic agent for effective diabetes management.

The induction of diabetes through alloxan administration occurs due to the selective destruction of pancreatic β‐cells, which are responsible for insulin production. This leads to impaired insulin secretion and the development of hyperglycemia, a hallmark of diabetes (Szkudelski 2001). Alloxan administration induced diabetes by disrupting metabolic balance and impairing kidney and liver functions. Specifically, it led to a reduction in body weight gain and an increase in the relative weights of the kidneys and liver. Additionally, alloxan caused elevated levels of blood glucose, cholesterol, triglycerides, urea, uric acid, creatinine, AST, and ALT. In diabetic conditions induced by alloxan, the rise in blood glucose is often accompanied by increased plasma cholesterol, triglycerides, and urea, reflecting widespread metabolic dysfunction (Cam et al. 1993; Pari and Saravanan 2002). The diabetogenic effects of alloxan are primarily linked to its selective cytotoxic action, which is mediated by the generation of reactive oxygen species (ROS). This oxidative stress leads to the destruction of a significant number of pancreatic β‐cells, resulting in reduced endogenous insulin secretion. Consequently, rats administered with alloxan rapidly develop hyperglycemia, further exacerbated by excessive hepatic glucose production. These combined effects contribute to the onset and progression of diabetes (Milagro and Martínez 2000). In diabetic rats induced by alloxan, there was a notable increase in plasma levels of urea, uric acid, and creatinine, all of which are critical biomarkers used to assess kidney function. These elevated levels indicate significant renal impairment, underscoring the adverse effects of diabetes on kidney health and the close relationship between chronic hyperglycemia and kidney dysfunction (Almdal and Vilstrup 1988; Burtis et al. 1996). The administration of the aqueous extract of *L. stoechas* effectively restored altered biochemical parameters toward normal levels. The observed elevation in plasma AST and ALT activities in diabetic rats is indicative of hepatic dysfunction, aligning with previous studies reporting liver damage and necrosis in diabetic conditions (Whitehead et al. 1999). The rise in transaminase levels is likely attributed to the release of these enzymes from injured liver cells (Larcan et al. 1979). However, treatment with *L. stoechas* aqueous extract normalized transaminase levels, highlighting its hepatoprotective effects. These results are consistent with research by Ohaeri et al. and Sebai et al., further confirming the protective potential of lavender against liver damage induced by alloxan (Sebai et al. 2013). More significantly, supplementation with *L. stoechas* prevented the alloxan‐induced reduction in plasma uric acid levels, a key endogenous water‐soluble antioxidant. This protective effect underscores the extract's ability to counteract oxidative stress and preserve antioxidant defenses, further highlighting its therapeutic potential in mitigating diabetes‐related complications (Estornell et al. 1994). These findings align with earlier studies, which have established a strong connection between diabetes and the overproduction of ROS. This interaction underscores the role of oxidative stress in the progression of diabetes and its complications, further validating the antioxidant properties of *L. stoechas* in addressing these effects (Al‐Azzawie and Alhamdani 2006; Sepici‐Dincel et al. 2007). Earlier research has consistently demonstrated that lavender extracts are abundant in phenolic compounds, which are known for their potent antioxidant and therapeutic properties. This richness in bioactive phenolics contributes to the extract's ability to combat oxidative stress and support its overall health benefits (Matos et al. 2009).

Alloxan‐induced diabetes causes marked metabolic disturbances, notably elevated total cholesterol and triglyceride levels, reflecting the dyslipidemia frequently associated with type 2 diabetes. This alteration is primarily attributed to insulin resistance and disrupted lipid metabolism, which lead to enhanced hepatic VLDL production and diminished lipid clearance (Bitzur et al. 2009). However, treatment with the aqueous extract of *L. stoechas* (AqLs) effectively reduced these elevated levels of total cholesterol and triglycerides, demonstrating its potential to counteract alloxan‐induced dyslipidemia. By normalizing lipid profiles.

According to Lipinski's Rule of Five (Laaraj, Choubbane, et al. 2024), a compound is considered a promising candidate if it meets at least four of the following criteria: a maximum of five hydrogen‐bond donors, no more than 10 hydrogen‐bond acceptors, an octanol–water partition coefficient (MLogP) of 5 or less, a polar surface area (PSA) not exceeding 140 Å^2^, and a molecular weight below 500 Da (Lipinski 2004). The majority of the compounds analyzed in our extract complied with these criteria, suggesting their good suitability for oral administration, with high intestinal absorption also being a key parameter influencing the bioavailability of orally administered molecules (Stillhart et al. 2020). Ferulic acid, 4‐hydroxybenzoic acid, cinnamic acid, and p‐coumaric acid have the potential to cross the BBB, which represents a crucial factor for their possible involvement in therapies targeting the central nervous system (Shimizu and Nakamori 2024). P‐glycoprotein (P‐gp), an efflux pump involved in limiting absorption and facilitating the elimination of xenobiotics, can influence the distribution and efficacy of active compounds (Kurbanova et al. 2024). The analyzed compounds were non‐substrates of P‐gp (PGP−). This characteristic is advantageous as it helps prevent premature elimination and ensures more stable and prolonged plasma concentrations. Hepatic metabolism, particularly interactions with cytochrome P450 enzymes, plays a central role in the biotransformation of exogenous molecules (Ellouz et al. 2024). The analysis conducted demonstrated that none of the identified compounds were inhibitors or substrates of CYP450 enzymes, notably CYP1A2. The absence of interaction with these enzymes suggests a reduced risk of altering the metabolism of concomitant drugs, thereby improving the safety profile of the studied compounds (Taşçıoğlu et al. 2021). These findings highlight the potential of the phytochemical compounds present in *L. stoechas* L. as promising candidates for the development of orally administrable drugs. The toxicity of a compound is closely linked to its classification, which is based on parameters such as LD_50_, effects on target organs, and mechanisms of action, allowing for the assessment of its risk to human health and the environment (Banerjee et al. 2018). Our compounds are classified between categories 4 and 6, which correspond to moderate to low toxicity, indicating a decreasing risk to human health (Banerjee and Ulker 2022). Furthermore, our compounds are considered non‐cytotoxic. This result, indicative of a low potential for cellular toxicity, reinforces their relevance for targeted therapeutic applications. They also exhibit good hepatic tolerance and are considered harmless to the immune system, suggesting an overall acceptable safety profile (Zwickl et al. 2022). These observations indicate that the *L. stoechas* extract has limited toxicity, which corroborates its observed safety in mice in our study (Elrherabi et al. 2024), further strengthening its potential for therapeutic applications (Zwickl et al. 2022).

## 5 Conclusion

The present study underscores the remarkable therapeutic potential of the aqueous extract of *L. stoechas* (AqLs) in managing diabetes and its associated complications. AqLs demonstrated significant antihyperglycemic effects by inhibiting carbohydrate‐digesting enzymes (α‐amylase and α‐glucosidase), reducing intestinal glucose absorption, and enhancing peripheral glucose uptake. Its antioxidant properties effectively mitigated oxidative stress, while its antiglycation and anti‐inflammatory activities further protected against diabetes‐related tissue damage. Additionally, AqLs improved lipid profiles by reducing plasma cholesterol and triglycerides, likely through the inhibition of pancreatic lipase and enhanced lipid metabolism. The extract also exhibited hepatoprotective and renoprotective effects, normalizing markers of liver and kidney function in alloxan‐induced diabetic rats. These multifaceted mechanisms, supported by the presence of bioactive phenolic compounds such as naringin, syringic acid, and cinnamic acid, underscore the potential of AqLs as a natural, multi‐targeted therapeutic agent for diabetes management.

Furthermore, in silico toxicity and ADME analyses revealed that the phytochemicals in AqLs generally exhibit favorable safety profiles and pharmacokinetic properties, making them promising candidates for oral drug development. However, further studies, including clinical trials, are warranted to validate these findings in humans and explore the full therapeutic potential of *L. stoechas* in combating diabetes and its complications. Overall, this research highlights the significant role of AqLs as a natural therapeutic option for addressing the complex metabolic and physiological challenges associated with diabetes.

## Author Contributions

**Amal Elrherabi:** conceptualization (equal), data curation (equal), formal analysis (equal), investigation (equal), methodology (equal), resources (equal), software (equal), validation (equal), writing – original draft (equal), writing – review and editing (equal). **Fahd A. Nasr:** funding acquisition (equal), investigation (equal), writing – review and editing (equal). **Ayoub Farihi:** conceptualization (equal), formal analysis (equal), methodology (equal), software (equal), validation (equal), writing – original draft (equal), writing – review and editing (equal). **Mohamed Bouhrim:** investigation (equal), validation (equal), visualization (equal), writing – review and editing (equal). **El Hassania Loukili:** writing – review and editing (equal). **Mounia Driouech:** methodology (equal), writing – review and editing (equal). **Nour elhouda Daoudi:** writing – review and editing (equal). **Mohammed Al‐zharani:** funding acquisition (equal), investigation (equal), writing – review and editing (equal). **Ashraf Ahmed Qurtam:** funding acquisition (equal), investigation (equal), writing – review and editing (equal). **Mohamed Bnouham:** project administration (equal), resources (equal), supervision (equal), visualization (equal), writing – review and editing (equal).

## Ethics Statement

The experimental protocol was reviewed and approved by the Ethics Committee for Laboratory Animals at the University of Lille (Approval Number: D5935010).

## Consent

All authors have read and approved the final version of the manuscript and consent to its publication.

## Conflicts of Interest

The authors declare no conflicts of interest.

## Data Availability

The original contributions presented in the study are included in the article. Further inquiries can be directed to the corresponding author.
